# The role of self-esteem and emotion regulation in the associations between childhood trauma and mental health in adulthood: a moderated mediation model

**DOI:** 10.1186/s12888-023-04719-7

**Published:** 2023-04-11

**Authors:** Cun Li, Peicai Fu, Minghuan Wang, Ye Xia, Caihong Hu, Mao Liu, Han Zhang, Xin Sheng, Yuan Yang

**Affiliations:** Department of Neurology and Psychiatry, Tongji Hospital, Tongji Medical College, Huazhong University of Science and Technology, Wuhan, 430030 China

**Keywords:** Childhood trauma, Mental health, Emotion regulation, Self-esteem, Moderation, mediation

## Abstract

**Background:**

High levels of childhood trauma (CT) have been observed in adults with mental health problems. Herein, we investigated whether self-esteem (SE) and emotion regulation strategies (cognitive reappraisal (CR) and expressive suppression (ES)) affect the association between CT and mental health in adulthood, including depression and anxiety symptoms.

**Methods:**

We performed a cross-sectional study of 6057 individuals (39.99% women, median age = 34 y), recruited across China via the internet, who completed the Patient Health Questionnaire-9 (PHQ-9), Generalized Anxiety Disorder-7 (GAD-7), Childhood Trauma Questionnaire (CTQ), Self-esteem Scale (SES), and Emotion Regulation Questionnaire (ERQ). Multivariate linear regression analysis and bias-corrected percentile bootstrap methodologies were used to assess the mediating effect of SE, and hierarchical regression analysis and subgroup approach were performed to examine the moderating effects of emotion regulation strategies.

**Results:**

After controlling for age and sex, we found that (1) SE mediated the associations between CT and depression symptoms in adulthood (indirect effect = 0.05, 95% confidence interval [CI]: 0.04–0.05, 36.2% mediated), and CT and anxiety symptoms in adulthood (indirect effect = 0.03, 95% CI: 0.03–0.04, 32.0% mediated); (2) CR moderated the association between CT and SE; and (3) ES moderated the association between of CT and mental health in adulthood via SE, and such that both the CT-SE and SE-mental health pathways were stronger when ES is high rather than low, resulting the indirect effect was stronger for high ES than for low ES.

**Conclusions:**

These findings suggested that SE plays a partially mediating role in the association between CT and mental health in adulthood. Furthermore, ES aggravated the negative effect of CT on mental health in adulthood via SE. Interventions such as emotional expression training may help reduce the detrimental effects of CT on mental health.

**Trial registration:**

The study was registered on http://www.chictr.org.cn/index.aspx and the registration number was ChiCTR2200059155.

**Supplementary Information:**

The online version contains supplementary material available at 10.1186/s12888-023-04719-7.

## Background

Epidemiological studies show that the rise of intense competition and survival pressure has resulted in a growing number of adults suffering from mental health problems [1]. Childhood trauma (CT), defined as emotional, physical, and sexual abuse, or emotional and physical neglect before the age of 16, has been extensively researched and established as a significant contributor to the development and deterioration of mental health in adulthood [2–6]. Studies have found that 28.9% of patients with psychiatric disorders have reported experiencing CT, and the effects of CT can persist throughout their lives [3]. A meta-analysis of the available literature suggests that experiencing any type of abuse may increase the risk of depression and anxiety symptoms in adulthood by over two-fold [7]. Given its impact on public health, it is important to further study the psychological symptomology of the effects of CT on mental health.

Bowlby J’s Attachment Theory states that adolescents develop coping mechanisms based on the influences of their parents and family environment to deal with stress, but experiences of adversity during this formative period can disturb the development of a healthy self-concept and secure attachments [8, 9]. Cumulative and severe exposure to adversity can lead to the development of poor self-concept, low self-esteem (SE), negative self-evaluation, and social withdrawal [10]. Prior research supports the theoretical link between the effects of CT and SE on mental health. A study of college students in Turkey found that SE acted as a mediator between CT and depression, anxiety, and stress symptoms in early adulthood [11]. In addition, three other studies conducted on children and adolescents showed that the negative influence of CT on mental health was mediated by lower SE [12–14]. These findings provide evidence for the important role of SE in the association between CT and mental health outcomes.

Previous literature has converged on the idea that cognition plays a crucial role in the development of depression and anxiety disorders [15]. Cognitive emotion regulation refers to attempts to respond to ongoing experiential demands with a range of emotions in a socially tolerable and sufficiently flexible manner [16, 17]. This can be characterized by two dimensions: cognitive reappraisal (CR) (adaptive emotion strategy, i.e., reinterpreting emotional events to alter the perceptions of their meaning and reduce emotional responses) and expressive suppression (ES) (maladaptive emotion strategy, i.e., suppressing outward expression of emotions that will occur or is occurring) [18]. Beck AT suggested that emotion regulation abilities are developed in early life [19] and adversity experiences in early life may make individuals more vulnerable to mental health difficulties in adulthood by altering stress response and emotion regulation systems [20], which play a key role in the development of mental health difficulties [21]. Cognitive theories put maladaptive emotion strategy at the core of depression and anxiety disorders.

Failure to effectively regulate emotions has been found to be strongly associated with the onset of mental health problems [22]. Empirical evidence provided support for the mediation role of cognitive emotion regulation strategies in the association between CT and mental health problems in various populations, including clinical sample [16], college student sample [23], children and adolescents [24, 25]. Individuals who have experienced CT are prone to subsequent emotion-regulation problems [26–29], ultimately leading to mental health problems, including depression and anxiety symptoms in later life [30, 31]. Besides the mediated effect, a growing body of research has revealed that emotion regulation moderates the association between CT and mental health problems in youth, and that the two interacting strategies, CR and ES, have different effects on mental health outcomes [32]. A lower use of CR and an increased use of ES were associated with high levels of depression and anxiety symptoms [33, 34]. In addition, Huang et al. revealed that Qi-stagnation constitution (QSC) partially mediated the association between CT on depression symptomology, and emotion dysregulation moderated the path between QSC and depression symptomology in college students [35].

Many studies have verified the mediating role of SE in the association between CT and mental health problems in college students [11], children and adolescents [12–14]. However, few studies have investigated it in adults. In addition, there is inconsistency in the role (as mediator or moderator) of emotion regulation strategies in previous research. CT may affect mental health problems via SE, but what role do emotion regulation strategies play in this process and under what circumstances do emotion regulation strategies influence the association between CT and mental health problems are remained to answer.

**Fig. 1:**
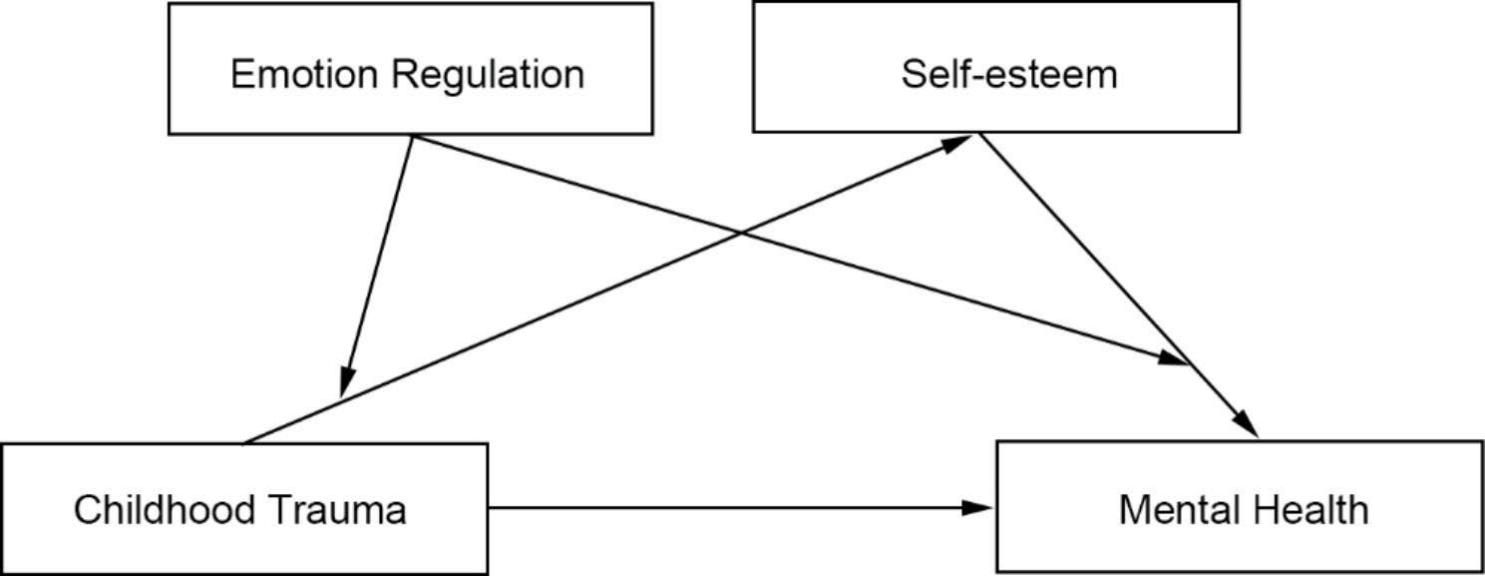
The conceptual moderated mediation model

To address the limitations in previous research, the current study aims to estimate the prevalence of lifetime CT reported by a large sample of healthy adults from across China, and the impact of SE and emotion regulation strategies on the association between CT and mental health outcomes. The study proposed the following hypotheses: (1) SE mediates the association between CT and mental health in adulthood; (2) CR moderates the association between CT and mental health in adulthood via SE, such that both the CT-SE and SE-mental health paths will be weaker when CR is high rather than low; and (3) ES will moderate the correlation between CT and mental health in adulthood via SE, such that both CT-SE and SE-mental health paths will be stronger when ES is high rather than low. As shown in Fig. 1, we constructed a moderated mediation model to elucidate the mechanisms underlying the association between CT and mental health, the mediating role of SE, and the moderating role of emotion regulation.

## Methods

### Participants and procedure

This cross-sectional study recruited participants who responded to the questionnaire delivered through a link on the Jingdong (JD) Insight platform (https://survey.jd.com/) across China from May 1, 2022, to May 30, 2022. The questionnaire contained questions on sociodemographic characteristics, anxiety, depression, and somatic symptoms, insomnia, emotion regulation, social support, SE, and CT. The inclusion criteria were: (1) 18 y of age or older; (2) Internet access via a smartphone or computer, and independent completion of the questionnaire; and (3) normal vision and hearing. The exclusion criteria were as follows: (1) severe or unstable physical disorder; (2) evidence of current or prior head injury, CNS disease, or other ICD-10 disorders; (3) history of substance abuse within six months before screening; (4) cognitive impairment; and (5) history of taking antipsychotics and long-acting injectable antipsychotics within the past two weeks. Eventually, 6057 individuals were included in the analysis. All data were collected online, and informed consent was obtained in accordance with the Declaration of Helsinki. This study was approved by the Ethics Committee of Tongji Hospital Affiliated Tongji Medical College, Huazhong University of Science and Technology (TJ-IRB20220408). The registration number for this trial was ChiCTR2200059155. The URL of the publicly accessible registered website is: http://www.chictr.org.cn/index.aspx.

## Measures

### Childhood trauma

CT was assessed using the Childhood Trauma Questionnaire (CTQ), an internationally accepted retrospective self-reported measure of five types of trauma experienced before age 16: emotional, physical, and sexual abuse, or emotional and physical neglect [36]. The CTQ consists of 28-item, rated on a 5-point frequency scale (1 = never true to 5 = very often true), and summed to yield a total score ranging from 5 to 25 for each type of trauma, with higher scores indicating greater severity [36]. The Chinese version of the CTQ has previously demonstrated good reliability and validity [37]. In the present study, the total CTQ score displayed good internal consistency (Cronbach’s α = 0.889).

### Cognitive emotion regulation strategies

The Emotional Regulation Questionnaire (ERQ) was used to assess regulation strategies in response to stressful events [18]. The ERQ has been widely used in the Chinese population with good reliability [38, 39] and it includes two dimensions, CR and ES [18], for which participants score how true (1 = not at all true, 7 = very true) each of 10 items are. In total, there are 6 items concerning the authenticity of CR (e.g., “I control my emotions by changing the way I think about the situation I am in”), while the other 4 items investigate the truth of ES (e.g., “I control my emotions by not expressing them”) [18]. The total scores of CR and ES were calculated separately, with higher scores indicating greater use to regulate emotions. In this study, the Cronbach’s α for CR and ES were 0.904 and 0.823, respectively.

### Self-esteem

SE was assessed using the Self-Esteem Scale (SES), which was developed by Rosenberg in 1965 [40] and is widely used in China to directly assess positive or negative feelings about oneself [41]. The SES consists of 10 items scored on a 4-point frequency scale (1 = strongly agree, 4 = strongly disagree), with a total score ranging from 10 to 40, with higher scores indicating greater SE. The Cronbach’s α coefficient of the SES in this study was 0.871.

### Depression and anxiety symptoms

Regarding mental health problems, we assessed participants’ depression and anxiety symptoms using the Patient Health Questionnaire-9 (PHQ-9) [42] and Generalized Anxiety Disorder-7 (GAD-7) [43], which has previously been validated in Chinese. On a 4-point Likert scale (1 = never, 4 = very often), participants reported how they felt or acted during the past two weeks. The mean was calculated across all items, with higher numbers indicating greater depression or anxiety symptoms. The PHQ-9 (Cronbach’s α = 0.927) and GAD-7 (Cronbach’s α = 0.937) scores showed good internal consistency.

### Statistical analyses

The general characteristics of the study population are represented by the number of cases and percentages of categorical variables and medians (interquartile range [IQR]) for continuous variables. Mann–Whitney U test and Pearson correlation analysis were used to compare the differences in terms of the severity of depression and anxiety symptoms.

All statistical analyses were conducted using SPSS v25.0 for Windows and SPSS AMOS v23. Structural equation modeling (SEM) is a powerful tool that is widely employed in various aspects of medicine [44]. The bias-corrected percentile bootstrap methodology was used in the present study, as it has been proven to have the greatest statistical performance for indirect effects in mediation models [45] and moderated mediation models [46].

Mediating effects were initially assessed by fitting a series of multivariate linear regression analyses [47, 48]. The AMOS macro for SPSS was used to investigate the mediating role of SE in the association between CT and depression and anxiety symptoms in adulthood. To investigate the indirect effect of SE, we performed percentile bootstrapping and bias-corrected percentile bootstrapping at a 95% confidence interval with 5,000 bootstrap samples and statistical significance set at a *p*-value < 0.05 [49]. We followed the recommendations of Preacher and Hayes (2008) [48] and calculated the confidence intervals of the lower and upper bounds to test whether the indirect effects were significant.

The moderated mediation effects of emotion regulation strategies were tested using path analytic methods accomplished through hierarchical regression analysis [50] and subgroup approach [51]. In all analyses, age and sex were entered as covariates [52]. First, we standardized all variables to reduce the potential effects of multicollinearity [50]. Second, we tested the first- and second-stage moderated mediated models outlined by MacKinnon [53], which can be divided into the following three steps. Step 1: The least squares technique was used with control variables entered as block 1 (age and sex). Step 2: The main effects are entered as block 2. Step 3: The moderators enter block 3. Finally, following the protocol of Edwards and Lambert (2007) [46], we calculated the simple effects under the mean (i.e., mean-centered), low (i.e., one SD below the mean), and high (i.e., one SD above the mean) levels of CR/ES. Differences in simple effects were computed by subtracting the effects for low CR/ES from those for high CR/ES, and indirect effects were calculated by multiplying the simple effects in both the first- and second-stage.

## Results

### Sample characteristics

A total of 6057 participants, comprising 2422 women (39.99%) and 3635 men (60.01%), were included in the analysis of the present study. The median (IQR) age of the participants was 34 (30–40) y. Pearson correlation analysis indicated that age was negatively associated with depression (r = -0.13, *p* < 0.001) and anxiety symptoms (r = -0.10, *p* < 0.001); however, the levels of depression and anxiety symptoms showed no significant difference based on sex (*p* > 0.05).

### Bivariate correlations

Pearson correlation analysis was used to test the bivariate correlations of all variables. As shown in Table 1, CT had a significantly negative association with SE (r = -0.41, *p* < 0.001) and a positive association with depression (r = 0.31, *p* < 0.001) and anxiety symptoms (r = 0.28, *p* < 0.001), while SE had a significantly negative correlation with depression (r = -0.35, *p* < 0.001) and anxiety symptoms (r = -0.30, *p* < 0.001). According to the associations among Cohen’s Standard, d, and r [54], all correlations showed small effects, except for the correlation between CT and SE, which showed a medium effect.

**Table 1:** Descriptive statistic and bivariate correlations between the variables

Variables	Mean	SD	1	2	3	4	5
1. Anxiety symptoms	3.12	4.05	-				
2. Depression symptoms	4.194	4.90	0.80^***^	-			
3. CT	38.46	11.67	0.28^***^	0.31^***^	-		
4. SE	31.09	5.49	-0.30^***^	-0.35^***^	-0.41^***^	-	
5. CR	26.77	7.53	-0.05^***^	-0.05^**^	-0.31^***^	0.28^***^	-
6. ES	14.31	4.82	0.09^***^	0.13^***^	-0.03	-0.05^***^	0.42^***^

*Note.* N = 6057. SD = standard deviation

CT = childhood trauma; SE = self-esteem; CR = cognitive reappraisal; ES = expressive suppression

** *p* < 0.01, *** *p* < 0.001

Multivariate linear regression analysis was performed to determine the associations between variables, and the results are presented in Additional file 1. After controlling for age and sex, higher CT was associated with lower SE (β = -0.19, *p* < 0.001), higher depression (β = 0.13, *p* < 0.001), and higher anxiety symptoms (β = 0.10, *p* < 0.001). Additionally, SE was negatively correlated with depression (β = -0.30, *p* < 0.001) and anxiety symptoms (β = -0.21, *p* < 0.001).

### Mediating effect of SE on the link between CT and mental health in adulthood

**Fig. 2:**
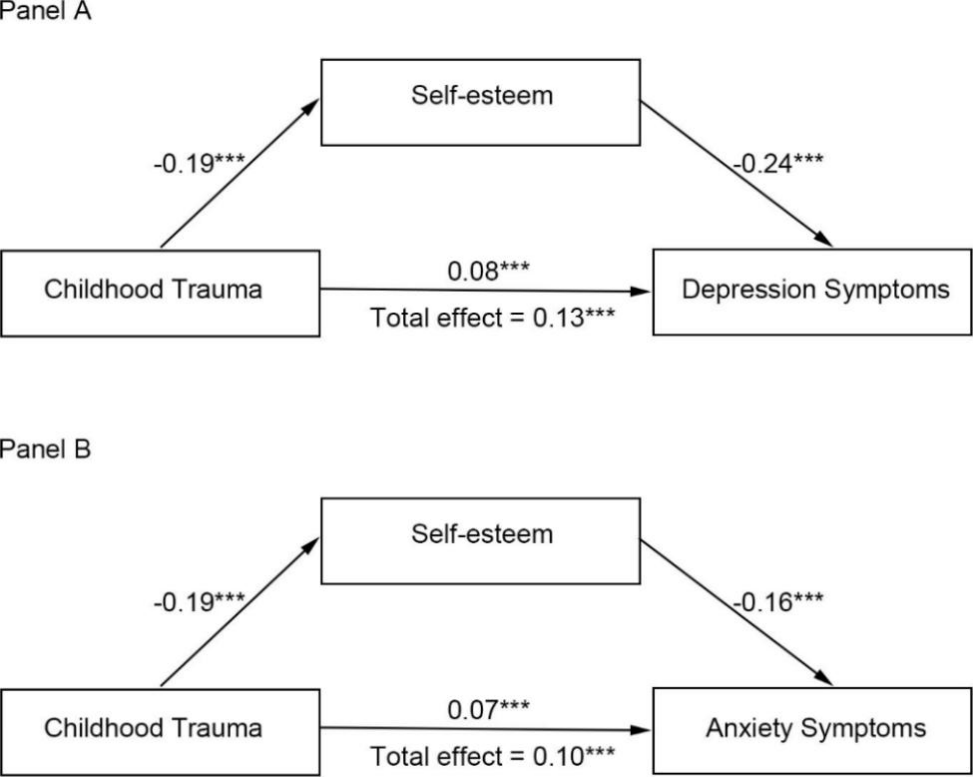
Self-esteem mediated the effects of childhood trauma on depression (Panel A) and anxiety (Panel B). *Note.* Unstandardized coefficients are presented. ^**^p < 0.01, ^***^p < 0.001

Two hypothetical mediation models were produced to test the mediating role of SE in the association between CT and mental health in adulthood in the context of SEM, for which the results are displayed in Table 2; Fig. 2. As shown in Table 2 and Panel A of Fig. 2, SE mediated the effect of CT on depression symptoms. Based on 5000 bootstrap resamples, the indirect path from CT to depression symptoms was significant (indirect effect = 0.05, 95% confidence interval [CI]:0.04–0.05, 36.2% mediated). Similarly, as shown in Table 2 and Panel B of Fig. 2, SE mediated the effect between CT and anxiety symptoms, and the indirect path from CT to anxiety symptoms was also significant (indirect effect = 0.03, 95% CI:0.03–0.04, 32.0% mediated). Taken together, these results suggest that SE mediated both the links between CT and depression symptoms as well as between CT and anxiety symptoms, suggesting that individuals with CT are more likely to have lower SE, which is associated with more depression and anxiety symptoms in adulthood.

**Table 2:** Unstandardized total, indirect and direct Effects of the two hypothetical models

Variables	Point estimated	Product of coefficients		Bootstrapping			
				Bias-Corrected 95% CI		Percentile 95% CI	
		SE	Z	Lower	Upper	Lower	Upper
**Unstandardized total effects**							
CT - SE - **depression** symptoms	0.13	0.01	18.57	0.12	0.14	0.12	0.14
CT – SE - anxiety symptoms	0.10	0.01	19.40	0.09	0.11	0.09	0.11
**Unstandardized indirect effects**							
CT - SE - **depression** symptoms	0.05	0.00	15.67	0.04	0.05	0.04	0.05
CT – SE - anxiety symptoms	0.03	0.00	15.50	0.03	0.04	0.03	0.04
**Unstandardized direct effects**							
CT - SE - **depression** symptoms	0.08	0.01	12.00	0.07	0.10	0.07	0.10
CT – SE - anxiety symptoms	0.07	0.01	11.00	0.05	0.08	0.05	0.08

*Note.* Unstandardized estimating of 5,000 bootstraps

CT = childhood trauma; SE = self-esteem

### Moderating role of emotion regulation strategies in the link between CT and mental health in adulthood via SE

As shown in Table 3, although CR moderated the CT-SE path (*p* < 0.001), it did not moderate either SE-depression symptoms (*p* > 0.05) or SE-anxiety symptoms (*p* > 0.05) paths. Additionally, there were no significant differences in the simple effects of the first- and second-stage as well as the indirect path (*p* > 0.05) (Table 4). In contrast, ES moderated not only the CT-SE path (*p* < 0.001), but also SE-depression symptoms (*p* < 0.001) and SE-anxiety symptoms (*p* < 0.001) (Table 5). Moreover, the moderated mediation effects of high and low levels of ES for the CT-SE-depression/anxiety symptom models were significantly different, as shown in Table 6.

**Table 3:** Coefficient estimates for the moderated mediation model for cognitive reappraisal

	First stage (dependent variable = SE)		Second stage (dependent variable = depression symptoms)		The second stage (dependent variable = anxiety symptoms)	
	B	t	B	t	B	t
***Step 1: Control variables***						
Age	0.02	10.97^***^	-0.02	-10.26^***^	-0.01	-7.94^***^
Sex	0.084	3.237^**^	-0.045	-1.727	-0.02	-0.79
R^2^	0.02		0.02		0.01	
F	64.05^***^		53.52^***^		31.61^***^	
***Sep 2: Main effects***						
Age	0.01	7.57^***^	-0.01	-5.78^***^	-0.01	-3.87^***^
Sex	-0.04	-1.82	0.01	0.32	0.03	1.25
CT	-0.35	-28.69^***^	0.22	16.52^***^	0.21	15.13^***^
CR	0.17	14.32^***^	0.11	8.44^***^	0.08	6.08^***^
SE			-0.28	-21.59^***^	-0.23	-17.05^***^
R^2^	0.20		0.17		0.13	
F	388.82^***^		251.43^***^		175.86^***^	
Incremental F	698.85^***^		376.72^***^		269.22^***^	
***Step3: Moderators***						
Age	0.01	7.67^***^	-0.01	-5.76^***^	-0.01	-3.86^***^
Sex	-0.04	-1.74	0.01	0.32	0.03	1.25
CT	-0.37	-29.07^***^	0.22	16.55^***^	0.21	15.07^***^
CR	0.18	14.85^***^	0.11	8.44^***^	0.08	6.08^***^
CT × CR	-0.05	-4.96^***^				
SE			-0.28	-21.42^***^	-0.23	-16.95^***^
SE ×CR			-0.01	-1.24	-0.01	-0.49
R^2^	0.21		0.17		0.13	
F	317.18^***^		209.80^***^		146.57^***^	
Incremental F	24.58^***^		1.53		0.24	

*Note.* N = 6057

The moderated mediation effect of cognitive reappraisal (CR) was tested by hierarchical regression, including the first- and second-stage moderated mediated model, each of which can be divided into the following three steps. For the first stage (dependent variable = self-esteem (SE)), age and sex were entered as a block in step 1, followed by main effects (childhood trauma (CT) and CR) in step 2, and moderators (CT × CR) in step 3. Then, for the second stage (dependent variable = depression/anxiety symptoms), age and sex were entered as a block in step 1, followed by main effects (CT, CR, and SE) in step 2, and moderators (SE × CR) in step 3

* *p* < 0.05, ** *p* < 0.01, *** *p* < 0.001

**Table 4:** Simple effect across the mean, low and high levels of cognitive reappraisal

Level	CT - SE - depression symptoms			CT - SE - anxiety symptoms		
	First stage	Second stage	Indirect effect	First stage	Second stage	Indirect effect
CR_low_	-0.16^**^	-0.30^***^	0.05^***^	-0.16^**^	-0.20^***^	0.03^***^
CR_Mean_	-0.18^***^	-0.24^**^	0.04^***^	-0.18^***^	-0.16^***^	0.04^**^
CR_High_	-0.19^***^	-0.25^***^	0.05^***^	-0.19^***^	-0.18^***^	0.04^***^
Difference between CR_High_ and CR_Low_	0.04	0.05	0.00	0.04	0.03	0.01

*Note.* N = 6057

The first- and second-stage simple effects for the mean, low, and high levels of cognitive reappraisal (CR) were calculated with coefficient estimates from Table 3. TAs were 0 (i.e., centered mean for CR), -1 (i.e., one SD below the mean), and 1 (i.e., one SD above the mean) for the mean, low and high levels of CR, respectively. Differences in simple effects were computed by subtracting the effects for low CR from those for high CR. Finally, the indirect effects were calculated by multiplying the simple effects in the first- and second stage. Significance tests for the indirect effects were based on bias-corrected confidence intervals derived from 5,000 bootstrapped samples

CT = Childhood Trauma; SE = Self-esteem. * *p* < 0.05, ** *p* < 0.01, *** *p* < 0.001

**Table 5:** Coefficient estimates for the moderated mediation model for expressive suppression

	First stage (dependent variable = SE)		Second stage (dependent variable = depression symptoms)		Second stage (dependent variable = anxiety symptoms)	
	B	t	B	t	B	t
***Step 1: Control variables***						
Age	0.02	10.97^***^	-0.02	-10.26^***^	-0.01	-7.94^***^
Sex	0.08	3.24^**^	-0.05	-1.73	-0.02	-0.79
R^2^	0.02		0.02		0.01	
F	64.03^***^		53.52^***^		31.61^***^	
***Sep 2: Main Effects***						
Age	0.01	7.60^***^	-0.01	-6.00^***^	-0.01	-4.02^***^
Sex	-0.04	-1.59	0.07	2.98^**^	0.08	3.09^**^
CT	-0.41	-34.26^***^	0.21	15.82^***^	0.20	14.66^***^
ES	-0.06	-5.38^***^	0.13	10.57^***^	0.09	7.33^***^
SE			-0.25	-19.69^***^	-0.21	-15.69^***^
R^2^	0.18		0.18		0.13	
F	335.30^***^		261.09^***^		179.68^***^	
Incremental F	594.03^***^		392.54^***^		275.52^***^	
***Step3: Moderators***						
Age	0.01	7.61^***^	-0.01	-5.85^***^	-0.01	-3.90^***^
Sex	-0.05	-1.85	0.07	2.66^**^	0.07	2.84^**^
CT	-0.40	-33.97^***^	0.21	16.07^***^	0.20	14.85^***^
ES	-0.07	-5.66^***^	0.13	10.59^***^	0.09	7.34^***^
CT × ES	0.03	3.19^**^				
SE			-0.25	-19.76^***^	-0.21	-15.74^***^
SE ×ES			-0.05	-4.73^***^	-0.04	-3.64^***^
R^2^	0.18		0.18		0.13	
F	270.68^***^		222.07^***^		152.24^***^	
Incremental F	10.16^**^		22.40^***^		13.24^***^	

*Note.* N = 6057

The moderated mediation effect of expressive suppression (ES) was tested by hierarchical regression, including the first- and second-stage moderated mediated model, each of which can be divided into the following three steps. For the first stage (dependent variable = self-esteem (SE)), age and sex were entered as a block in step 1, followed by main effects (childhood trauma (CT) and ES) in step 2, and moderators (CT × ES) in step 3. Then, for the second stage (dependent variable = depression/anxiety symptoms), age and sex were entered as a block in step 1, followed by main effects (CT, ES, and SE) in step 2, and moderators (SE × ES) in step 3

* *p* < 0.05, ** *p* < 0.01, *** *p* < 0.001

**Table 6:** Simple effect across the mean, low and high levels of expression suppression

Level	CT - SE - depression symptoms			CT - SE - anxiety symptoms		
	First stage	Second stage	Indirect effect	First stage	Second stage	Indirect effect
ES_Low_	-0.22^**^	-0.17^***^	0.04^***^	-0.22^**^	-0.12^***^	0.03^***^
ES_Mean_	-0.19^***^	-0.23^**^	0.04^***^	-0.19^***^	-0.15^***^	0.03^***^
ES_High_	-0.18^***^	-0.34^***^	0.06^***^	-0.18^***^	-0.25^***^	0.04^***^
Difference between ES_High_ and ES_Low_	0.04	0.17^**^	0.03^*^	0.04	0.13^**^	0.02^*^

*Note.* N = 6057. The first- and second-stage simple effects for the mean, low, and high levels of expressive suppression (ES) were calculated with coefficient estimates from Table 5. TAs were 0 (i.e., centered mean for ES), -1 (i.e., one SD below the mean), and 1 (i.e., one SD above the mean) for the mean, low and high levels of ES, respectively. Differences in simple effects were computed by subtracting the effects for low ES from those for high ES. Finally, the indirect effects were calculated by multiplying the simple effects in the first- and second stage. Significance tests for the indirect effects were based on bias-corrected confidence intervals derived from 5,000 bootstrapped samples

CT = Childhood Trauma; SE = Self-esteem

* *p* < 0.05, ** *p* < 0.01, *** *p* < 0.001

For the CT–SE–depression symptoms model, the first- and second- stage simple effects for low ES were − 0.22 (*p* < 0.01) and − 0.17 (*p* < 0.001), resulting in an indirect effect of 0.04 (*p* < 0.001). For high ES, the first- and second-stage simple effects were − 0.18 (*p* < 0.001) and − 0.34 (*p* < 0.001), yielding an indirect effect of 0.06 (*p* < 0.001). Furthermore, the results of the comparison between low and high ES indicated that the first-stage simple effect did not differ significantly (0.04, *p* > 0.05); however, the second-stage simple effect was stronger for high ES than for low ES (0.17, *p* < 0.01), and the indirect effect comparison between them was also statistically different (0.03, *p* < 0.05).

For the CT–SE–anxiety symptoms model, the first- and second-stage simple effects for low ES were − 0.22 (*p* < 0.01) and − 0.12 (*p* < 0.001). Thus, the indirect effect for low ES was 0.03 (*p* < 0.001). For high ES, the first- and second-stage simple effects were − 0.18 (*p* < 0.001) and − 0.25 (*p* < 0.001), respectively. Hence, the indirect effect for high ES was 0.04 (*p* < 0.001). In addition, the results of the comparison between low and high ES suggest that the first-stage simple effects for high and low ES did not differ significantly (0.04, *p* > 0.05); however, the second-stage simple effect was stronger for high ES than for low ES (0.13, *p* < 0.01). Finally, the indirect effect comparison was found to be statistically different (0.02, *p* < 0.05).

### Sensitivity analysis

A sensitivity analysis was conducted to ensure that the above findings were not influenced by potential confounders including age and sex. For this purpose, all analyses were repeated without the above covariates, as shown in Additional files 2 and 3. There were no meaningful changes in the significance or magnitude of the above findings, indicating their robustness.

## Discussion

While several studies have indicated the mediating role of SE between CT and mental health problems, and more studies have addressed the impact of emotion regulation strategies on mental health trajectory, few have linked both in adulthood. The present study therefore presents evidence for the mediating role of SE in a large sample population and finds a moderating effect of ES on the association between CT and mental health in adulthood via SE.

In line with Hypothesis 1, our results showed that SE partially mediated the association between CT and depression and anxiety symptoms in adulthood. According to attachment theory, exposure to abuse in childhood can lead to diminished perceptions of oneself, as well as emotional problems that threaten future psychological health [55]. In other words, abused children are less likely to perceive themselves as valuable and important and have less ability to deal with emotional problems compared to those who do not experience abuse [56, 57]. Our results are consistent with past research showing that university students with traumatic experiences have lower total SES scores [11, 58]. In addition, as a state of being self-satisfied without considering oneself inferior or superior, a decline in SE may induce mental health problems [58]. A meta-analysis of longitudinal studies covering 77 studies on depression and 18 on anxiety symptoms confirmed the negative effect of SE on depression and anxiety symptoms levels [59]. Moreover, another study indicated that low SE predicted subsequent levels of depression in adolescence and young adulthood [60]. Taken together with the results of the present study, this suggests a potential mechanism through which CT affects mental health in adulthood.

In addition, the results of the hierarchical regression analysis showed that ES moderated the CT-SE, SE-depression symptoms, and SE-anxiety symptom pathways, whereas CR moderated only the CT-SE pathway. However, the comparison between low and high CR/ES indicated no significant differences in the simple effects of the CT-SE path. The inconsistent results of the hierarchical regression analysis and subgroup approach may be due to the following reasons. First, research conducted within each subgroup has a lower statistical power than the full sample [61]. Second, studies using subgroup approach often form subgroups by dichotomizing the continuous moderating variables. Compared to hierarchical regression analysis, this approach results in the loss of information, and often leads to biased parameter estimates, further reducing statistical power [62, 63]. Finally, as shown in Additional files 4 and 5, both ES and CR are not normally distributed, and it is not appropriate to divide groups simply using mean-centered data. Taken together, these results partially support hypothesis 2 and fully support hypothesis 3.

As an adaptive emotion regulation strategy, CR diminishes negative effects by reinterpreting one’s thinking about the situation that causes negative emotions [22]. Although both CR and ES have mood-reducing functions, individuals with high levels of CR may be more likely to respond to adverse events with problem-focused coping rather than avoidance, and this problem-focused coping strategy is usually effective, especially in Western cultures [64]. In addition, another study suggested that CR could buffer the negative effects of CT on mental health in patients with depression disorder [16]. However, in the present study, we found that CR moderated the association between CT and SE, whereas no moderated mediation effect of CR on the association between CT and mental health in adulthood was found. We therefore surmised that these inconsistent results may be attributed to the relatively small sample size of previous studies, as well as the differences in ethnicity [64].

Furthermore, the results demonstrated that ES moderated the association between CT and mental health in adulthood via SE, such that the indirect path was stronger when ES was high rather than low, indicating that ES aggravated the negative effect of CT on mental health in adulthood via SE. ES generally refers to altering the way a person’s behavior reacts to events that elicit emotions by suppressing their outward expression [65]. As a reaction-focused emotion regulation strategy [18], the use of ES appears to be less effective at reducing negative emotions [65], which in turn is associated with negative mental health outcomes [66]. Thus, in line with our results, ES is generally considered a maladaptive emotional regulation strategy and a risk factor for depression and anxiety symptoms, whereas CR is considered an adaptive strategy, and is associated with lower levels of depression and anxiety symptoms [66]. The more often ES was used to regulate emotions in the face of negative emotional stimuli, the lower the SE of those who experienced CT, which is related to depression and anxiety symptoms in adulthood. Although individuals temporarily inhibit the outward expression of negative emotions with good intentions, there is generally no evidence to suggest that it can help alleviate negative emotional experiences from CT in the long term [18, 52, 67].

Moreover, several studies have focused on the moderating role of culture and ethnicity in the association between ES and mental health outcomes, demonstrating that ES is generally detrimental to mental health outcomes across cultures. Although Asian individuals have fewer negative outcomes correlated with ES compared to Western individuals [68–70], ES nevertheless remains highly prevalent among the Chinese population, as emotional control and restraint are considered virtues in Chinese culture [18, 68]. In the present study, we also found that the use of ES in individuals who experienced CT aggravated the negative effects on mental health in adulthood via SE, which is in line with previous research on emotion regulation in Asian youth [71]. Thus, individuals may benefit from developing healthy emotion regulation profiles that include less suppression during adulthood.

### Strengths and limitations

To the best of our knowledge, this study is the first to investigate the underlying moderated mediation mechanisms of emotion regulation strategies on mental health among those who experienced CT. These results have several practical implications. For example, children should be given more care to avoid psychological symptoms in adulthood, and intervention programs should consider the mediating role of SE between CT and mental health in adulthood. In addition, though emotional control and restraint are considered virtues in Chinese culture, our study suggests that ES is harmful to mental health because it can aggravate the negative effect of CT on mental health in adulthood via SE. Encouraging emotional expression and release may reduce the impact of CT on mental health.

However, several limitations must be addressed in future research. First, the information was collected via online surveys, which are more subjective than the results of a structured interview, which is limited by its brief and nondiagnostic nature. Second, CT recall bias may be possible, which may affect the reliability of retrospective assessments of traumatic experiences in children. Finally, as a cross-sectional study, it was difficult to establish a causal association between the variables, and further longitudinal studies are recommended to determine the causal association between them.

## Conclusion

In summary, the results of this study suggest that SE partially mediates the association between CT and mental health in adulthood. Furthermore, ES aggravates the negative effect of CT on mental health in adulthood via SE. Future interventions and policy recommendations aiming to reduce the detrimental effects of CT should consider the role of emotion regulation.

## Electronic supplementary material

Below is the link to the electronic supplementary material.

Supplementary Material 1: *Additional file 1* Multiple linear regression results for testing the association between CT/SE and mental health. *Additional file 2* Coefficient estimates for the moderated mediation model for cognitive reappraisal. *Additional file 3* Coefficient estimates for the moderated mediation model for expressive suppression. *Additional file 4* The distribution of cognitive reappraisal. *Additional file 5* The distribution of expressive suppression.

## Data Availability

The data that support the findings of this study are available on request from the corresponding author. The data are not publicly available due to privacy or ethical restrictions.

## Abbreviations

CT: Childhood trauma
SE: Self-esteem
CR: Cognitive reappraisal
ES: Expressive suppression
QSC: Qi-stagnation constitution
JD: Jingdong
CTQ: Childhood Trauma Questionnaire
ERQ: Emotional Regulation Questionnaire
SES: Self-Esteem Scale
PHQ-9: Patient Health Questionnaire-9
GAD-7: Generalized Anxiety Disorder-7
IQR: Interquartile range
SEM: Structural equation modeling
SD: Standard deviation
CI: Confidence interval.
