# Supplementary material for: The role of self-esteem and emotion regulation in the associations between childhood trauma and mental health in adulthood: a moderated mediation model
Source: BMC Psychiatry. 2023 Apr 11;23:241. doi: 10.1186/s12888-023-04719-7 (PMC10091572; doi:10.1186/s12888-023-04719-7)
Supplement: Supplementary file 1 — Supplementary Material 1: Additional file 1 Multiple linear regression results for testing the association between CT/SE and mental health. Additional file 2 Coefficient estimates for the moderated mediation model for cognitive reappraisal. Additional file 3 Coefficient estimates for the moderated mediation model for expressive suppression. Additional file 4 The distribution of cognitive reappraisal. Additional file 5 The distribution of expressive suppression. [file 12888_2023_4719_MOESM1_ESM.docx]

Additional file 1 Multiple linear regression results for testing the association between CT/SE and mental health.

|  | Depression symptoms | | | | Anxiety symptoms | | | |
| --- | --- | --- | --- | --- | --- | --- | --- | --- |
|  | Unadjusted | | Adjusted^%^ | | Unadjusted | | Adjusted^%^ | |
|  | β | 95%CI | β | 95%CI | β | 95%CI | β | 95%CI |
| CT | 0.13^***^ | 0.12~0.14 | 0.13^***^ | 0.12~0.14 | 0.10^***^ | 0.09~0.10 | 0.10^***^ | 0.09~0.10 |
| SE | -0.32^***^ | -0.34~-0.29 | -0.30^***^ | -0.33~-0.28 | -0.22^***^ | -0.24~-0.20 | -0.21^***^ | -0.23~-0.20 |

*Note.* CI = confidence interval; CT = childhood trauma; SE = self-esteem.

^%^Adjusted for age and sex.

* *p* < 0.05, ** *p* < 0.01, *** *p* < 0.001.

Additional file 2 Coefficient estimates for the moderated mediation model for cognitive reappraisal

|  | First stage (dependent variable = SE) | | Second stage (dependent variable = depression symptoms) | | Second stage (dependent variable = anxiety symptoms) | |
| --- | --- | --- | --- | --- | --- | --- |
|  | B | t | B | t | B | t |
| ***Step 1: Main effects*** | | | | | | |
| CT | -0.36 | -29.57^***^ | 0.22 | 16.98^***^ | 0.21 | 15.39^***^ |
| CR | 0.17 | 14.17^***^ | 0.11 | 8.56^***^ | 0.08 | 6.26^***^ |
| SE |  |  | -0.29 | -22.23^***^ | -0.24 | -17.53^***^ |
| R^2^ | 0.20  739.13^***^ | | 0.17  405.64^***^ | | 0.12  286.68^***^ | |
| F |  |  |  |  |  |  |
| ***Step2: Moderators*** | | | | | | |
| CT | -0.37 | -29.88^***^ | 0.23 | 17.02^***^ | 0.21 | 15.34^***^ |
| CR | 0.18 | 14.70^***^ | 0.11 | 8.56^***^ | 0.08 | 6.26^***^ |
| CT × CR | -0.05 | -4.83^***^ |  |  |  |  |
| SE |  |  | -0.29 | -22.03^***^ | -0.24 | -17.42^***^ |
| SE ×CR |  |  | -0.02 | -1.33 | -0.01 | -0.55 |
| R^2^ | 0.20  502.35^***^  23.33^***^ | | 0.17  304.71^***^  1.77 | | 0.12  215.06^***^  0.31 | |
| F |  |  |  |  |  |  |
| Incremental F |  |  |  |  |  |  |

*Note.* N = 6057.

The moderated mediation effects of cognitive reappraisal (CR) were tested by hierarchical regression, including the first and second-stage moderated mediated models. As there was no control variable, each stage can be divided into the following two steps. For the first stage (dependent variable = self-esteem (SE)), the main effects (childhood trauma (CT) and CR entered as a block in step 1, followed by the moderators (CT × CR) in step 2. Then, for the second stage (dependent variable = depression/anxiety symptoms), the main effects (CT, CR, and SE) were entered as a block in step 1, followed by the moderators (SE × CR) in step 2.

* *p* < 0.05, ** *p* < 0.01, *** *p* < 0.001.

Additional file 3 Coefficient estimates for the moderated mediation model for expressive suppression

|  | First stage (dependent variable = SE) | | Second stage (dependent variable = depression symptoms) | | Second stage (dependent variable = anxiety symptoms) | |
| --- | --- | --- | --- | --- | --- | --- |
|  | B | t | B | t | B | t |
| ***Step 1: Main effects*** | | | | | | |
| CT | -0.41 | -35.35^***^ | 0.21 | 15.98^***^ | 0.19 | 14.69^***^ |
| ES | -0.06 | -5.03^***^ | 0.12 | 10.03^***^ | 0.08 | 6.78^***^ |
| SE |  |  | -0.26 | -20.39^***^ | -0.21 | -16.22^***^ |
| R^2^ | 0.17  633.44^***^ | | 0.17  416.44^***^ | | 0.13  289.24^***^ | |
| F |  |  |  |  |  |  |
| ***Step2: Moderators*** | | | | | | |
| CT | -0.41 | -35.01^***^ | 0.21 | 16.29^***^ | 0.20 | 14.91^***^ |
| ES | -0.06 | -5.27^***^ | 0.12 | 10.12^***^ | 0.08 | 6.84^***^ |
| CT × ES | 0.03 | 2.99^**^ |  |  |  |  |
| SE |  |  | -0.26 | -20.44^***^ | -0.21 | -16.25^***^ |
| SE ×ES |  |  | -0.06 | -5.13^***^ | -0.05 | -3.99^***^ |
| R^2^ | 0.17  425.835^***^  8.962^***^ | | 0.18  320.222^***^  26.343^***^ | | 0.13  221.436^***^  15.879^***^ | |
| F |  |  |  |  |  |  |
| Incremental F |  |  |  |  |  |  |

Note. N = 6057.

The moderated mediation effects of expressive suppression (ES) were tested by hierarchical regression, including the first- and second-stage moderated mediated models. As there was no control variable, each stage can be divided into the following two steps. For the first stage (dependent variable = self-esteem (SE)), the main effects (childhood trauma (CT) and ES entered as a block in step 1, followed by the moderators (CT ×ES) in step 2. Then, for the second stage (dependent variable = depression/anxiety symptoms), the main effects (CT, ES, and SE) were entered as a block in step 1, followed by the moderators (SE × ES) in step 2.

* *p* < 0.05, ** *p* < 0.01, *** *p* < 0.001.


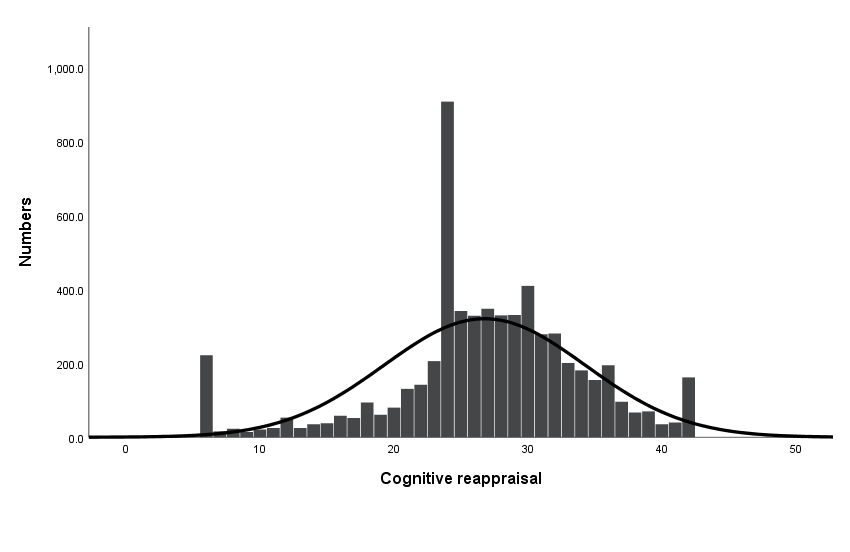


Additional file 4 The distribution of cognitive reappraisal


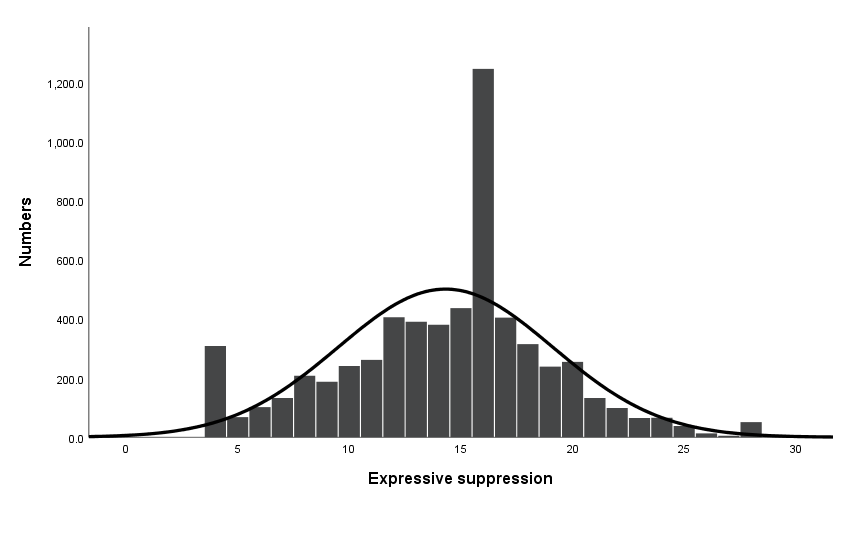


Additional file 5 The distribution of expressive suppression
